# Association between the systemic immune-inflammation index and prognosis in patients with stroke: a meta-analysis of cohort studies

**DOI:** 10.3389/fneur.2026.1760467

**Published:** 2026-02-06

**Authors:** Ziliang Zhang, Zhihong Huang, Zhenzhou Guo, Yan Shi, Jinhua Qiu

**Affiliations:** 1 Department of Neurology, Xinfeng County People’s Hospital, Ganzhou, Jiangxi, China; 2 Department of Neurology, Ganzhou People’s Hospital, Ganzhou, Jiangxi, China

**Keywords:** stroke, systemic immune-inflammation index, SII, clinical outcome, meta-analysis

## Abstract

**Introduction:**

The systemic immune-inflammation index (SII) is a newly recognized biomarker of inflammation. Although several studies have suggested that SII may aid in diagnosis of stroke and in predicting treatment outcomes, the findings remain inconsistent, and its relationship with clinical prognosis is still unclear. Therefore, we conducted a comprehensive systematic review and meta-analysis to explore the relationship between SII and clinical outcomes in patients with stroke.

**Methods:**

We systematically searched four databases (PubMed, Embase, Cochrane Library, and Web of Science). The study adhered strictly to PRISMA guidelines. We assessed the risk of bias across the included studies using the Newcastle–Ottawa Scale. Key outcome indicators included poor functional outcome (modified Rankin Scale, mRS ≥ 2), mortality, stroke severity (National Institutes of Health Stroke Scale, NIHSS >4), and intracranial hemorrhage.

**Results:**

A total of 11 cohort studies comprising 24,922 patients with stroke were included. Our results demonstrated that elevated SII was strongly linked to increased mortality (OR = 1.58, 95% CI: 1.23–2.02; *p* = 0.0003) and poor functional outcome (mRS ≥ 2) (OR = 2.03, 95% CI: 1.63–2.52; *p* = 0.0001). However, elevated SII was not associated with NIHSS >4 (OR = 3.40, 95% CI: 2.02–5.71; *p* = 0.80), nor with intracranial hemorrhage (OR = 2.41, 95% CI: 1.59–3.66; *p* = 0.35).

**Conclusion:**

SII appears to have potential value in predicting stroke prognosis and may help clinicians assess outcomes by calculating patients’ SII levels. Nevertheless, given the limitations of the available evidence, further research is needed to clarify its practical clinical utility. Larger samples and multicenter clinical trials are required to obtain more robust conclusion.

**Systematic review registration:**

https://www.crd.york.ac.uk/PROSPERO/view/CRD420251163979, identifier PROSPERO (CRD420251163979).

## 1 Introduction

Cerebrovascular diseases are currently the second leading cause of death and disability worldwide, exhibiting marked geographical, age-related, and risk-factor–specific patterns (1). Recent WHO data indicate that cerebrovascular disease affects over 15 million people annually across the globe, resulting in over 6 million deaths. In China alone, the annual number of newly diagnosed stroke cases exceeds 3.8 million, and the prevalence continues to rise at an average rate of 8.3% per year (2–4). From the perspective of population distribution, the incidence increases exponentially after the age of 55. Individuals aged 75 years and older have a more than tenfold higher risk compared with those aged 45–54 years, and men consistently exhibit a greater incidence than women (5). Of particular concern is that modifiable risk factors—including hypertension, diabetes, dyslipidemia, smoking, and obesity—account for more than 80% of cases. Moreover, the proportion of younger patients developing stroke due to unhealthy lifestyle behaviors has been steadily increasing, imposing substantial physical, psychological, and economic burdens on patients and their families (6).

Stroke is commonly classified into ischemic and hemorrhagic forms, with ischemic events comprising about 87% of all cases. In clinical practice, management follows the principle of “subtype-specific treatment with time as the priority.” For ischemic stroke, the central therapeutic goal is rapid restoration of cerebral perfusion. Intravenous thrombolysis is recommended within 4.5 h of onset, and mechanical thrombectomy can be performed within 12 h (7). Long-term management requires antiplatelet therapy, statins, and stringent control of blood pressure, blood glucose, and other comorbid conditions. For hemorrhagic stroke, the primary objectives are reducing intracranial pressure and preventing rebleeding. During the acute stage, blood pressure should be promptly lowered to ≤140/90 mmHg. Patients with a hematoma volume greater than 30 mL or those at risk of brain herniation require timely surgical evacuation—either via craniotomy or minimally invasive approaches (8). Prognosis after stroke is influenced by treatment timing, disease severity, underlying comorbidities, and rehabilitation interventions. Evidence indicates that ischemic stroke patients who receive standardized therapy within the first hour achieve favorable outcomes in more than 70% of cases. In contrast, acute-phase mortality in hemorrhagic stroke ranges from 30 to 40%, and only about 20% of survivors regain independent living abilities. Patients with diabetes or atrial fibrillation tend to have poorer outcomes and exhibit a 2–3-fold increased risk of recurrence (9). Comprehensive secondary prevention combined with long-term rehabilitation can effectively improve outcomes, with limb-function recovery rates increasing by up to 50% and recurrence reduced by approximately 40% (10). Conversely, unhealthy lifestyle behaviors—such as smoking and excessive alcohol consumption—may elevate the risk of recurrence by 2–5-fold. Early detection, accurate assessment of stroke severity, and timely management of risk factors are therefore crucial for improving long-term prognosis.

With the increasing focus on elucidating the pathophysiological mechanisms of stroke, accumulating evidence suggests that its development is strongly influenced by inflammatory mechanisms. Suppressing the activation and infiltration of inflammatory cells has been shown to mitigate neurological injury (11, 12). Consequently, immunological therapies targeting inflammation could provide effective approaches for enhancing treatment outcomes in stroke patients. Identifying efficient and reliable biomarkers to predict prognosis is therefore of great importance, as it may help reduce adverse clinical events (13, 14). The systemic immune-inflammation index (SII) has emerged as a novel inflammatory biomarker, calculated as platelet count × (neutrophil/lymphocyte ratio) (15). It indicates the balance between immune and inflammatory responses and has demonstrated strong prognostic value across a variety of diseases (16–18). Recent studies have also proposed that SII may serve as a useful predictor in stroke. For example, Ma et al. (19) reported that patients with acute ischemic stroke who experienced poor outcomes had significantly higher SII levels compared with those with favorable outcomes, and elevated SII was identified as an independent predictor of unfavorable 90-day prognosis. Similarly, Arslan and sahin (20) stratified patients according to 28-day mortality and functional outcomes, showing that SII had significant predictive utility for both endpoints. Nevertheless, the relationship between SII and clinical outcomes following stroke remains contentious, and existing findings are inconsistent. In 2022, Huang et al. (21) published a meta-analysis addressing this association; however, the included studies were highly heterogeneous, consisting mainly of retrospective designs such as cohort and cross-sectional studies. The literature search was also limited to only 469 records, and the pooled results were potentially affected by methodological confounders. Meanwhile, numerous new studies examining SII and stroke outcomes have been published in recent years. In light of this growing evidence base, the present meta-analysis aims to build upon previous work by restricting inclusion strictly to cohort studies and incorporating the most up-to-date research. Our objective is to more comprehensively evaluate the association between SII and clinical outcomes in patients with stroke.

## 2 Materials and methods

### 2.1 Literature search

This review was conducted in accordance with the PRISMA 2020 guidelines, and the study protocol was registered in PROSPERO (CRD420251163979). Ziliang Zhang and Zhihong Huang developed the search strategy, formulating subject terms and keywords to search PubMed, Embase, Web of Science, and the Cochrane Library. The search encompassed studies from each database’s inception to September 30, 2025. The search terms and detailed search strategies were as follows: ((“Stroke”[Mesh]) OR ((((Strokes) OR (Cerebral Stroke)) OR (CVA)) OR (Acute Cerebrovascular Accident))) AND ((systemic immune-inflammation index) OR (SII)) (shown in Supplementary Table S1).

### 2.2 Study selection

Eligible studies were required to meet the following criteria:

1. patients diagnosed with stroke according to the 2023 Chinese Guidelines for the Diagnosis and Treatment of Acute Ischemic Stroke;
2. studies that primarily assessed the prognostic impact of SII in stroke;
3. studies that reported odds ratios (ORs) with 95% confidence intervals (CIs), either directly or calculable from the available data;
4. studies in which patients were categorized into high-SII and low-SII groups based on predefined cutoff values;
5. fully published articles.

The exclusion criteria were as follows:

1. reviews, commentaries, conference abstracts, case reports, and letters;
2. studies lacking sufficient information to calculate ORs and 95% CIs;
3. studies without complete or extractable data;
4. duplicate publications or studies with overlapping datasets.

Titles and abstracts of all retrieved records were independently screened by two investigators (Ziliang Zhang and Zhihong Huang), after which full-text articles were examined for eligibility. Any conflicts in judgment were addressed through discussion until consensus was reached.

### 2.3 Data extraction

Data extraction was performed independently by two investigators, Ziliang Zhang and Zhihong Huang. Any discrepancies were resolved through discussion among all co-authors. The extracted information included the first author’s name, year of publication, country (study location), study design, sample size, patient age, study duration, treatment methods, reported mortality outcomes, specific NIHSS scores, mRS scores, timing of SII measurement, cutoff values, follow-up duration, and the effect estimates for SII (odds ratios with 95% confidence intervals). When the same study reported multiple sets of parallel data, we extracted them separately or split them for independent extraction.

### 2.4 Quality assessment

Methodological quality was assessed using the Newcastle–Ottawa Scale (NOS), covering the domains of selection, comparability, and outcome. The highest possible score was 9 points. Studies scoring between 7 and 9 points were considered to be of high methodological quality.

### 2.5 Statistical analysis

Pooled odds ratios (ORs) with corresponding 95% confidence intervals (CIs) were calculated to evaluate the prognostic value of SII in patients with stroke. Heterogeneity among studies was assessed using Cochran’s Q test and Higgins’ I^2^ statistic. Significant heterogeneity was considered present when I^2^ > 50% or *p* < 0.1, in which case a random-effects model was applied for all analyses.

Sensitivity analyses and subgroup analyses were conducted to examine the robustness of the findings and to explore potential sources of heterogeneity. Publication bias was assessed using Egger’s test. Statistical significance was set at *p* < 0.05. Stata 18.0 and Review Manager 5.4 were used for all data analyses.

## 3 Results

### 3.1 Study characteristics

**Figure 1:**
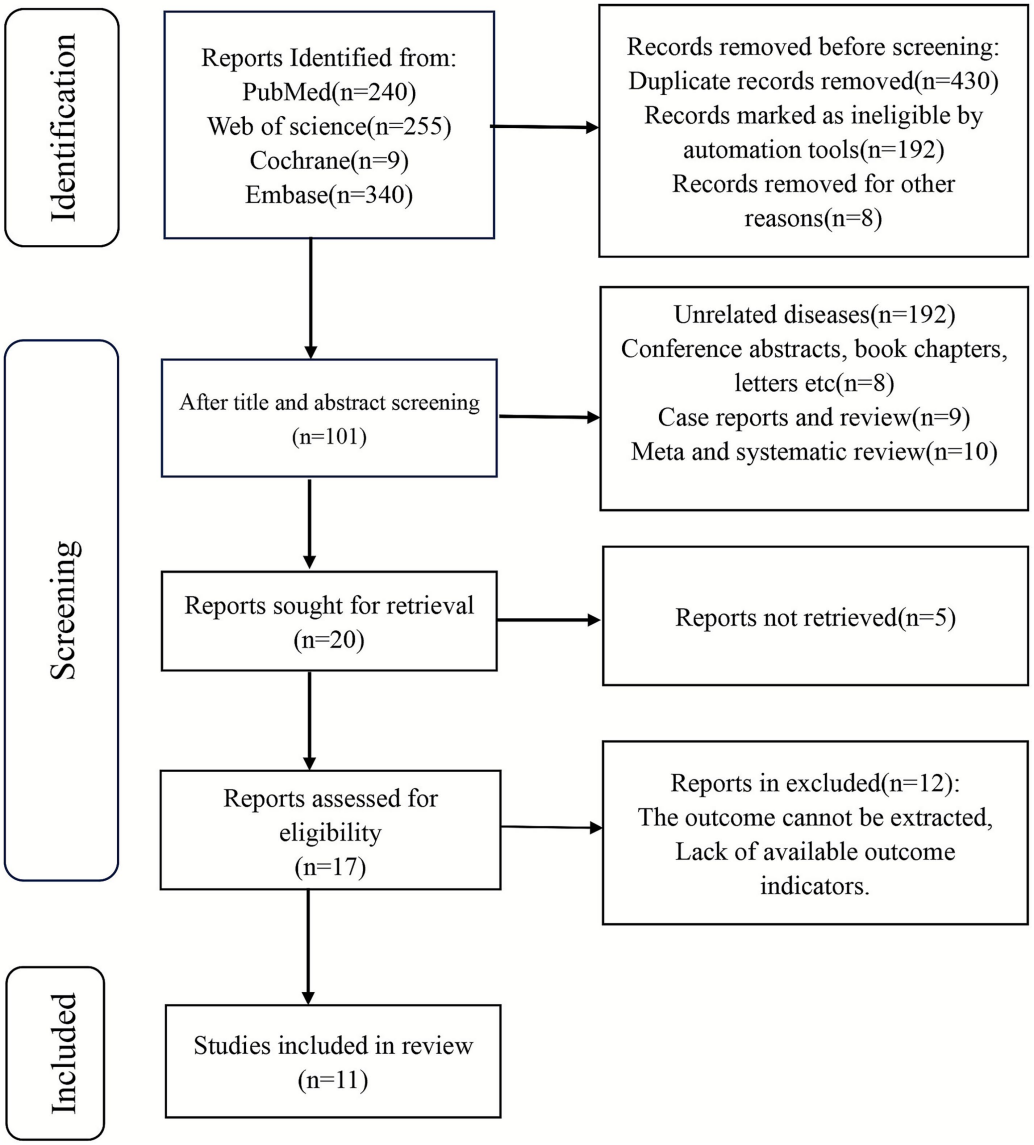
PRISMA flowchart of included studies.

In the initial search, 844 records were identified across the databases. After removing 430 duplicates, 414 studies remained for title and abstract screening, during which 387 were excluded. Eleven articles were retrieved for full-text review. Among the included studies, four contained parallel datasets, yielding a total of 18 comparison groups and 24,922 patients for the final meta-analysis (Figure 1).

Of the 11 eligible studies, two were conducted in the United States and South Korea, while the remaining nine were carried out in China. The included studies consisted entirely of cohort designs and were published in English between 2022 and 2025. One study involved patients treated with mechanical thrombectomy, whereas the other 10 focused on intravenous thrombolysis. In each study, patients were categorized into high-SII and low-SII groups for analysis. SII was measured at baseline in all studies and re-evaluated after treatment. The prognostic value of SII was assessed based on mortality, NIHSS scores, mRS scores, and the occurrence of intracranial hemorrhage. The characteristics of the included studies are summarized in Table 1.

**Table 1:** Basic characteristics of the included literature.

Author	Year	Sources of patients	Study design	Participants (n)	Age (y) (mean ± SD)	Male (%)	Type of stroke	Time of blood sample	Type of intervention	Cut off of SII	Primary end points	Follow up (d)	NOS
Jun et al.	2023	American	Retrospective cohort	693	68.02(55.69, 78.13)	334	AIS	On arrival in the emergency room	MT	NA	Mortality	30-day	7
Ao et al. (a)	2025	China	Retrospective cohort	81	70.80 ± 12.64	46	AIS	Within 24 h of admission	MT	658	Mortality	90-day	9
Ao et al. (b)	2025	China	Retrospective cohort	81	70.74 ± 11.01	35	AIS	Within 24 h of admission	MT	1,087	Mortality	90-day	9
Ao et al. (c)	2025	China	Retrospective cohort	81	69.54 ± 11.32	44	AIS	Within 24 h of admission	MT	2,659	Mortality	90-day	9
Suwen et al. (a)	2024	China	Retrospective cohort	1,268	67 (59–76)	835	AIS	Within 24 h of admission	rt-PA-IVT	369	Mortality NIHSS	1 year	8
Suwen et al. (b)	2024	China	Retrospective cohort	1,268	67 (59–76)	835	AIS	Within 24 h of admission	rt-PA-IVT	531.27	Mortality NIHSS	1 year	8
Suwen et al. (c)	2024	China	Retrospective cohort	1,268	67 (59–76)	835	AIS	Within 24 h of admission	rt-PA-IVT	772.29	Mortality NIHSS	1 year	8
Kadiyan et al.	2025	China	Retrospective cohort	541	74.23 ± 10.46	322	AIS	Within 24 h of admission	rt-PA-IVT	NA	Mortality	1 week	7
Nan et al. (a)	2022	China	Retrospective cohort	9,107	62.7 ± 10.7	6,343	AIS	Within 24 h of admission	NA	366	Mortality NIHSS	1 year	8
Nan et al. (b)	2022	China	Retrospective cohort	9,107	61.4 ± 10.9	6,343	AIS	Within24 h of admission	NA	533	Mortality NIHSS	1 year	8
Nan et al. (c)	2022	China	Retrospective cohort	9,107	61.5 ± 11.3	634	AIS	Within 24 h of admission	NA	799	Mortality NIHSS	1 year	8
Yiyun et al.	2021	China	Retrospective cohort	216	68.5 (59.25–76)	136	AIS	On arrival in the emergency room	rt-PA-IVT	545.14	NIHSS	90-day	7
Yun-Xiang et al.	2022	China	Retrospective cohort	208	63.3 ± 11.3	143	AIS	Within 24 h of admission	NA	802.8	NIHSS mRS	90-day	9
Zhang et al.	2023	China	Retrospective cohort	245	59 (51–70)	171	AIS	On admission	NA	NA	mRs	6-month	6
Yuan et al.	2022	China	Retrospective cohort	127	69 (56, 78)	69	AIS	On admission	rt-PA	NA	HT	72 h	7
Yuan et al.	2022	China	Retrospective cohort	126	67 (54, 76)	62	AIS	On admission	rt-PA	NA	HT	72 h	7
Yi et al.	2021	China	Retrospective cohort	310	65.0 ± 11.4	225	AIS	Within 24 h of admission	rt-PA	653.65	NIHSS	24 h	8
Ho et al.	2021	Korea	Retrospective cohort	195	72.6 (11.7)	108	AIS	Within 24 h of admission	rt-PA	NA	mRs	90-day	8

### 3.2 Study quality

All 11 studies scored between 7 and 8 on the Newcastle–Ottawa Scale, indicating high methodological quality (see Supplementary Table S2).

### 3.3 Meta-analysis results

#### 3.3.1 SII and mortality

**Figure 2:**
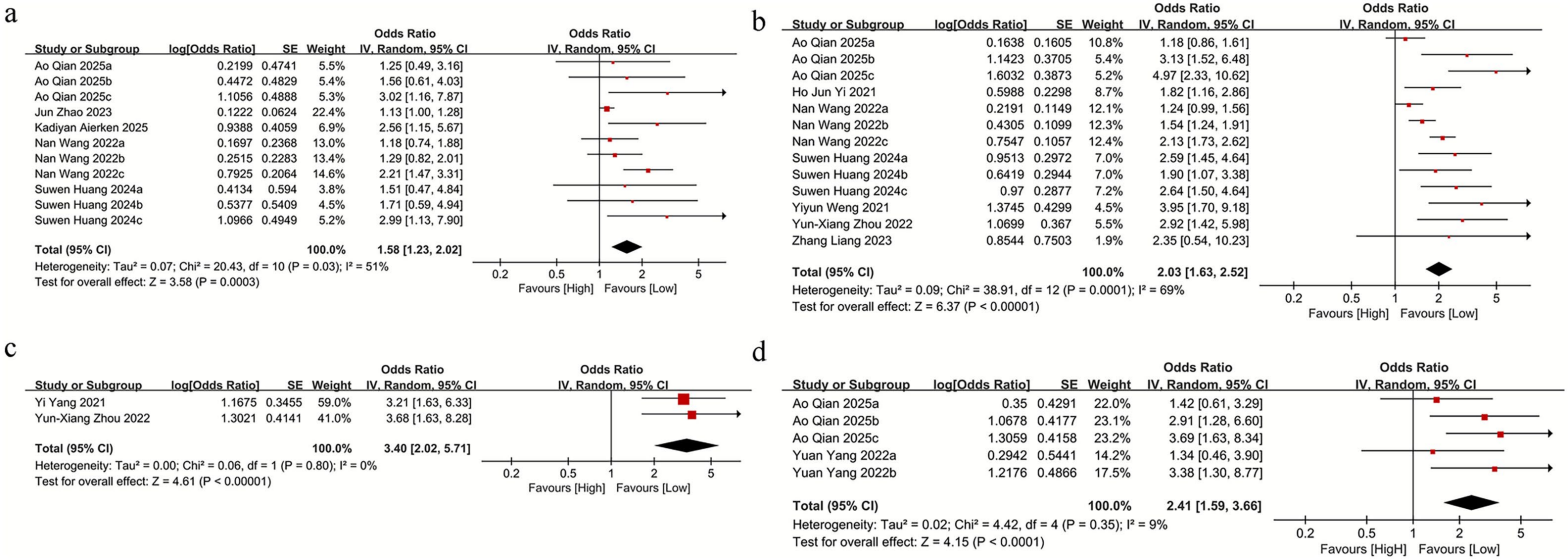
**(a)** The mortality between the high SII and low SII groups; **(b)** SII and mRS score≥2 between the high SII and low SII groups; **(c)** SII and NIHSS score>4 between the high SII and low SII groups; **(d)** SII and intracranial hemorrhage between the high SII and low SII groups.

We assessed the relationship between SII and mortality across five studies, three of which included parallel datasets, yielding a total of 11 comparison groups. Owing to significant heterogeneity among the included studies (*I*^2^ = 51%), a random-effects model was applied. The pooled analysis indicated that higher SII levels were strongly linked to increased mortality (OR = 1.58, 95% CI: 1.23–2.02; *p* = 0.0003) (Figure 2a). Subgroup analyses according to sample size, country, age, and SII cutoff values revealed that the association between SII and mortality was not statistically significant in the subgroup with an SII cutoff ≥500 (OR = 1.23, 95% CI: 0.80–1.89; *p* = 0.36) (Supplementary Table S3), whereas significant associations were observed in all other subgroups. Heterogeneity analyses indicated that sample size, study location, age, and SII cutoff were the primary contributors to between-study variability.

#### 3.3.2 SII and mRS score≥2

For the association between SII and mRS scores, seven studies were included, three of which contained parallel datasets, resulting in 13 comparison groups. Given the substantial heterogeneity among these studies (*I*^2^ = 69%), a random-effects model was applied. The pooled results demonstrated a significant association between elevated SII and poor functional outcome (mRS ≥ 2) (OR = 2.03, 95% CI: 1.63–2.52; *p* = 0.0001) (Figure 2b). Subgroup analyses were performed according to sample size, study location, age, and SII cutoff values. The findings indicated that elevated SII remained significantly associated with mRS ≥ 2 across all subgroup categories (Supplementary Table S2).

#### 3.3.3 SII and NIHSS score>4

For the association between SII and NIHSS scores, two studies were included. Owing to the absence of heterogeneity among these studies (*I*^2^ = 0%), a random-effects model was applied. The pooled analysis indicated that higher SII levels were not significantly linked to NIHSS >4 (OR = 3.40, 95% CI: 2.02–5.71; *p* = 0.80) (Figure 2c).

#### 3.3.4 SII and intracranial hemorrhage

For the association between SII and intracranial hemorrhage (ICH), two studies were included, both of which reported parallel datasets, yielding a total of five comparison groups. Given the presence of heterogeneity among these studies (*I*^2^ = 9%), a random-effects model was applied. The pooled analysis indicated that elevated SII was not significantly associated with ICH (OR = 2.41, 95% CI: 1.59–3.66; *p* = 0.35) (Figure 2d). Similarly, subgroup analyses based on sample size, study location, age, and SII cutoff values indicated no significant link between SII and ICH across any subgroup categories (Supplementary Table S2).

### 3.4 Sensitivity analysis

**Figure 3:**
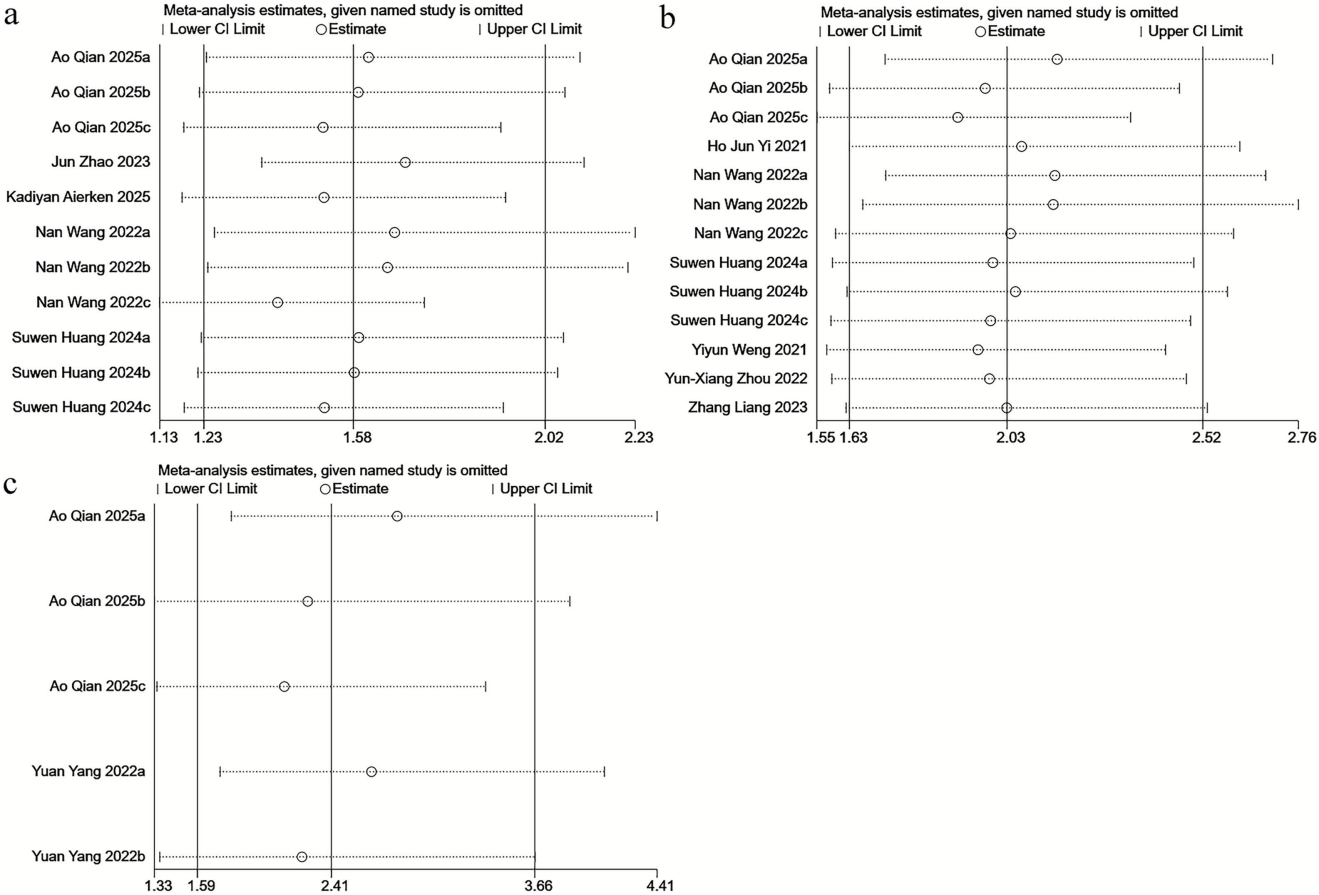
Sensitivity analysis of **(a)** mortality, **(b)** mRS, and **(c)** intracranial hemorrhage.

Similarly, sensitivity analyses were conducted to evaluate the robustness of the results and to examine the consistency of the clinical implications of baseline SII. The results showed that sequential exclusion of each individual study did not materially alter the effect sizes for mortality (Figure 3a), mRS ≥ 2 (Figure 3b), or intracranial hemorrhage (Figure 3c). These findings indicate that no single study exerted an undue influence on the overall association between SII and stroke outcomes, thereby confirming the stability and reliability of our results.

### 3.5 Publication bias

**Figure 4:**
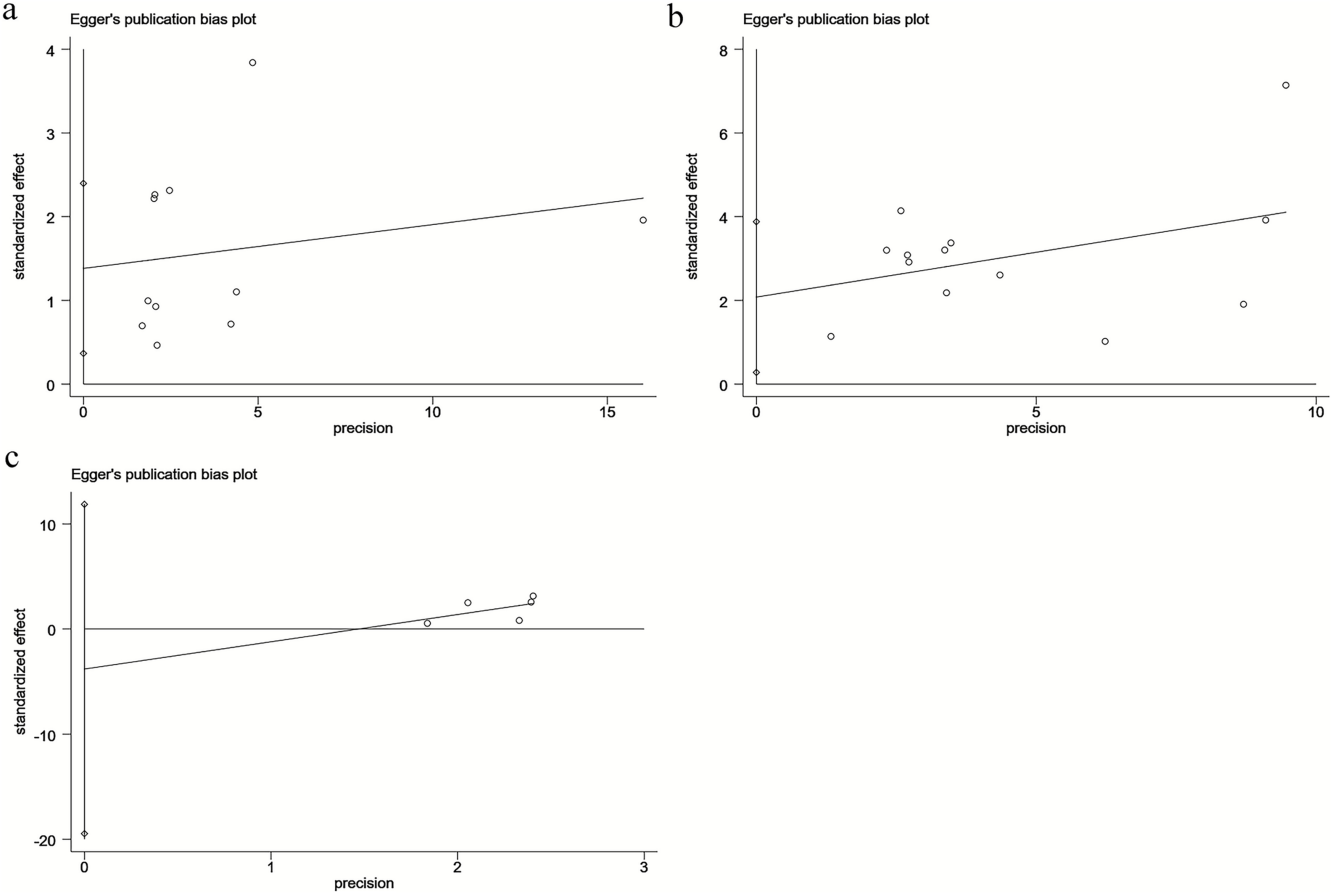
Egger’s analysis of **(a)** mortality, **(b)** mRS, and **(c)** intracranial hemorrhage.

Publication bias was assessed using Egger’s test for mortality, mRS ≥ 2, and intracranial hemorrhage. The results indicated potential publication bias for mortality (Egger’s *p* = 0.013) (Figure 4a) and mRS ≥ 2 (Egger’s *p* = 0.027) (Figure 4b), whereas no significant publication bias was observed for intracranial hemorrhage (Egger’s *p* = 0.496) (Figure 4c). However, due to the limited number of available studies, publication bias could not be evaluated for the remaining outcomes.

## 4 Discussion

Our study demonstrated that SII was strongly associated with mortality and mRS ≥ 2 in patients with stroke, whereas no significant association was observed with NIHSS >4 or intracranial hemorrhage. Sensitivity analyses for mortality, mRS ≥ 2, and intracranial hemorrhage showed that the effect sizes remained stable within the original ranges after sequential exclusion of each individual study, indicating that no single study disproportionately influenced the overall associations. These findings support the robustness and reliability of our results. In addition, Egger’s tests revealed no significant publication bias for the association between SII and intracranial hemorrhage, although publication bias was detected for mortality and mRS ≥ 2. It is noteworthy that our findings share several similarities with the meta-analysis published by Yong-Wei et al. in 2022 (21). Both studies demonstrated that elevated SII was significantly associated with poor functional outcomes and higher mortality in patients with stroke. Consistent patterns were observed in which patients with unfavorable outcomes, those who died, and those with moderate to severe stroke exhibited markedly higher SII levels compared with individuals with favorable outcomes, survivors, and patients with mild stroke. However, our study extends previous evidence by providing a more refined evaluation of the prognostic significance of SII for mortality and poor outcomes. In particular, subgroup analyses for mortality, mRS ≥ 2, and intracranial hemorrhage revealed that SII lost its predictive value when the cutoff was ≥500, whereas values <500 retained strong prognostic significance. These findings further clarify that SII demonstrates meaningful predictive ability only within a certain threshold range, thereby offering valuable guidance for clinical decision-making. Importantly, our analysis also included newly published studies that investigated the association between SII and intracranial hemorrhage, and we found no significant relationship between SII levels and the occurrence of ICH. By incorporating a larger sample size and minimizing bias—especially through the inclusion of the most recent evidence from the past 3 years—our study provides more robust and up-to-date insights into the clinical utility of SII in predicting outcomes after stroke.

At the same time, the prognostic value of SII in stroke appears to exhibit marked dose-dependent characteristics. However, substantial heterogeneity exists across studies regarding the selection of SII cutoff values, and these thresholds often lack unified scientific justification. Such inconsistencies not only limit the clinical translation and practical application of SII but also highlight gaps in our current understanding of the molecular and pathological mechanisms underlying stroke at different disease stages. The biological processes driving these variations remain largely unexplored and represent an important area for future research (12, 22–24).

In recent years, studies on the pathophysiological mechanisms of stroke have shown that stroke leads to cellular rupture and the release of large amounts of damage-associated molecular patterns (DAMPs) (25). These DAMPs are recognized by pattern recognition receptors on the surface of immune cells in the circulation, thereby initiating both innate and adaptive immune responses (26). Under intense inflammatory signaling, regulatory T cells and Th2 cells in the peripheral blood undergo programmed cell death (27). Meanwhile, a subset of activated T and B cells is “recruited” to the perilesional brain tissue or to the draining lymph nodes to clear necrotic debris and potential pathogens, resulting in a marked reduction in the total lymphocyte count in the peripheral circulation.

Concurrently, DAMPs released from brain tissue and tissue factor exposed by damaged vascular endothelium continuously activate neutrophils, thereby triggering the NETosis cell death program and the assembly of neutrophil extracellular traps (NETs) (28). The formation of NETs can directly damage vascular endothelial cells and further recruit additional neutrophils, creating a self-amplifying positive inflammatory feedback loop. Histones within NETs can directly activate platelets, inducing the release of granule contents, upregulation of adhesion molecules such as P-selectin, and cleavage of fibrinogen, thereby activating coagulation factor FXII and initiating the coagulation cascade (29). Platelets activated by NETs specifically bind to neutrophils via interactions between platelet surface P-selectin and neutrophil P-selectin glycoprotein ligand-1 (PSGL-1), forming stable neutrophil–platelet aggregates (NPs) (30). The resulting thrombi directly obstruct blood flow, leading to more extensive cerebral necrosis.

Based on the findings of this study, we speculate that an SII cutoff value of ≥500 may represent an optimal threshold for initiating clinical intervention. This is particularly relevant for patients with acute stroke. For example, in cases complicated by infection, timely initiation of anti-infective therapy may be warranted. In patients with large-vessel occlusion, post-reperfusion “inflammatory rebound” may occur after endovascular treatment, and anti-inflammatory agents such as edaravone dexborneol may help mitigate this response (31). In addition, in patients with a hypercoagulable state, anticoagulant therapy (e.g., low-molecular-weight heparin) may reduce platelet activity. These interventions directly influence components of the SII calculation, thereby contributing to therapeutic benefits.

Varjú et al. (32), in a study of patients with atrial fibrillation–related acute ischemic stroke undergoing intravenous thrombolysis, demonstrated that elevated SII remained an independent predictor of mortality even after adjustment for age, comorbidities, and other confounders. In a large-scale cohort study, Wang et al. (33) reported that SII strongly predicted long-term stroke recurrence and all-cause mortality, independent of conventional risk factors such as hypertension and diabetes. Similarly, Varjú et al. (32) found that higher SII levels were significantly linked to poor functional outcomes and increased mortality at both 90-day and 1-year follow-up in patients with AIS. Collectively, these studies consistently indicate that SII outperforms other inflammation-related markers—including the neutrophil-to-lymphocyte ratio, platelet-to-lymphocyte ratio, and lymphocyte-to-monocyte ratio—because it simultaneously integrates neutrophil, platelet, and lymphocyte counts. By capturing the immune–inflammation–thrombosis axis central to stroke pathophysiology, SII demonstrates particular value in atrial fibrillation–related stroke, aligning with previous findings that highlight the pivotal role of systemic inflammation in stroke (34, 35). Mechanistically, SII reflects dysregulation across three major cell types involved in stroke pathology. Elevated neutrophils promote oxidative stress, blood–brain barrier disruption, and thrombosis; decreased lymphocyte counts indicate suppressed immune function; and activated platelets contribute to both thrombogenesis and inflammation. Together, these processes synergistically drive adverse clinical outcomes. Moreover, blood–brain barrier disruption and endothelial injury further increase the risk of hemorrhagic transformation and mortality (36–38).

Recent studies have shown that SII differs significantly across clinical outcome groups and correlates closely with stroke severity, further underscoring its potential as a tool for clinical stratification (39). In current practice, stroke assessment largely relies on subjective or complex measures such as the NIHSS score and neuroimaging findings. In contrast, SII—derived from routine complete blood count parameters—offers several advantages, including low cost, reproducibility, and rapid availability. In the emergency setting, SII can be used for early risk assessment, enabling rapid identification of high-risk patients who may require more intensive anti-inflammatory treatment or optimized blood pressure management to reduce the risk of hemorrhagic transformation. Moreover, dynamic monitoring of SII may help evaluate treatment response; declining SII levels after intervention suggest controlled inflammation and a favorable prognosis, whereas persistently elevated levels indicate the need for therapeutic adjustment (40). It is important to note that the prognostic value of inflammatory biomarkers appears to be highly stroke-subtype specific. Zhao et al. (41), using the mixed stroke cohort from the MIMIC-III database, reported that NLR, the neutrophil-to-albumin ratio, and the red cell distribution width–to–albumin ratio were strong predictors of 30-day mortality in patients with hemorrhagic stroke, but were not associated with mortality in ischemic stroke. This discrepancy reflects the distinct inflammatory triggers underlying the two subtypes: hemorrhagic stroke induces an immediate and intense systemic inflammatory response driven by acute tissue destruction, hematoma expansion, and blood–brain barrier disruption; whereas ischemic stroke involves reperfusion injury and microvascular occlusion, generating a more transient or less readily captured inflammatory signal—particularly when measured at a single ICU admission time point. Further supporting this subtype-specific pattern, Qian et al. (42) demonstrated in AIS patients undergoing mechanical thrombectomy that SII independently predicted hemorrhagic transformation and malignant cerebral edema. They also showed that mild hypothermia reduced these risks among patients with high SII levels, suggesting that biomarker selection and interpretation must be tailored to stroke subtype and clinical context. Moreover, work by Huang et al. revealed that sharp increases or decreases in SII during hospitalization carried greater prognostic value than baseline SII alone, challenging the traditional paradigm of relying on single-time-point biomarker measurements. In Zhao’s cohort, the lack of association between SII and mortality in ischemic stroke may therefore be attributable to reliance solely on admission-time SII measurements, which fail to capture the dynamic inflammatory evolution described by Huang et al. In ischemic stroke, inflammation follows a temporal pattern—initial pro-inflammatory injury followed by a reparative phase—and dysregulation at either stage may lead to poor outcomes. Continuous SII monitoring may allow timely detection of these transitions and help identify patients at heightened risk of inflammatory deterioration.

Despite providing valuable insights, several limitations should be acknowledged in this meta-analysis. First, the majority of all eligible studies included in this analysis were conducted in Asian populations, specifically in China and South Korea. Therefore, our conclusions should be interpreted within this geographic context, and caution is required when extrapolating these findings to populations in Europe, Africa, the Americas, or other regions. In particular, for the outcome measures NIHSS >4 (2 studies) and intracranial hemorrhage (ICH; 2 studies), the number of included studies was limited, resulting in a low strength of evidence; consequently, additional studies are required to further validate the association between SII and prognosis in patients with stroke. Further studies in diverse populations are required to validate the association between SII and stroke prognosis globally. Second, most of the included studies were retrospective rather than prospective in design. The retrospective nature may introduce confounding factors and reduce the reliability of the results. Selection bias and unmeasured confounders—such as incomplete documentation of comorbidities or variability in treatment strategies—are unavoidable. Although the included studies performed multivariable adjustments for common confounders such as age, sex, hypertension, and diabetes, the possibility of residual confounding cannot be ruled out. Third, current research on the relationship between SII and clinical outcomes in stroke remains limited by relatively small sample sizes, homogeneous study populations, and short follow-up durations, making it difficult to assess the long-term prognostic value of SII. More importantly, mechanistic studies exploring how SII influences stroke outcomes are still scarce. While we hypothesize that SII may affect stroke progression through dysregulation of the inflammation–immunity–thrombosis axis, this assumption has not yet been validated by experimental data, nor has the interaction between SII and other inflammatory biomarkers been clarified. These knowledge gaps restrict our understanding of the biological functions of SII and hinder the development of SII-based targeted therapeutic strategies. Additionally, there was inconsistency in the cutoff values used to define high SII across the included studies, with thresholds generally falling above or below 500. This variability represents another limitation. Nonetheless, SII demonstrated independent predictive value for stroke outcomes across multiple analyses.

Based on the current state of research and the existing gaps in the field, we believe that future studies should focus on several key directions to advance both the mechanistic understanding and clinical translation of SII in stroke. First, large-scale, multicenter, prospective clinical studies are needed to further validate the association between SII and both short-term and long-term outcomes in patients with stroke, and to determine the optimal SII cutoff value. Establishing standardized study protocols will help reduce heterogeneity across studies and provide more robust evidence for the clinical application of SII. Moreover, future research should include more diverse populations—across different ethnicities, geographic regions, and stroke subtypes—to assess the generalizability of SII and improve the external validity of the findings. Second, mechanistic investigations using stroke animal models are warranted to examine temporal changes in SII-related immune cell populations and functions in both brain tissue and peripheral blood. Such studies may elucidate the specific molecular pathways through which SII regulates the pathological progression of stroke. In parallel, cellular experiments should explore the interactions between SII-related immune cells and neuronal or endothelial cells to clarify how SII influences blood–brain barrier integrity, neuronal injury, and repair processes. Third, integrating SII with NIHSS scores and infarct volume to construct composite predictive models may help identify patients at high risk of hemorrhagic transformation and support the development of individualized treatment strategies for clinicians. Finally, interventional clinical studies based on SII are warranted. Given the variability across studies in measurement time points (e.g., at admission or within 24 h) and in methods used to determine cutoff values (e.g., ROC curve–based thresholds or median-based definitions), future research should standardize baseline assessment timing and establish unified cutoff criteria through multicenter studies. In addition, particular emphasis should be placed on evaluating whether targeted anti-inflammatory therapies can improve prognosis in patients with stroke by assessing their impact on clinical outcomes.

## 5 Conclusion

In conclusion, this meta-analysis demonstrates that elevated SII is associated with poorer clinical outcomes in patients with stroke. These findings suggest that SII may serve as an independent and clinically informative prognostic biomarker, aiding in treatment decision-making and potentially guiding early therapeutic intervention. However, given the methodological limitations of the included studies, additional high-quality evidence is needed. Well-designed, multicenter, prospective studies with larger sample sizes and diverse populations are essential to validate our findings across different ethnic and geographic groups and to further establish the prognostic utility of SII in stroke care.

## Data Availability

The original contributions presented in the study are included in the article/Supplementary material, further inquiries can be directed to the corresponding author.
