# Supplementary material for: Association between the systemic immune-inflammation index and prognosis in patients with stroke: a meta-analysis of cohort studies
Source: Front Neurol. 2026 Feb 6;17:1760467. doi: 10.3389/fneur.2026.1760467 (PMC12920226; doi:10.3389/fneur.2026.1760467)
Supplement: Supplementary file 1 [file Table_1.docx]

**Supplementary materials**

TableS1 Literature search strategy

Pubmed-240

(("Stroke"[Mesh]) OR ((((Strokes) OR (Cerebral Stroke)) OR (CVA)) OR (Acute Cerebrovascular Accident))) AND ((systemic immune-inflammation index) OR (SII))

Embase-340

((Stroke or (Strokes or Cerebral Stroke or CVA or Acute Cerebrovascular Accident)) and (systemic immune-inflammation index or SII)).af.

Cochrane-9

((Stroke or (Strokes or Cerebral Stroke or CVA or Acute Cerebrovascular Accident)) and (systemic immune-inflammation index or SII)).af.

WOS-255

((Stroke) OR ((((Strokes) OR (Cerebral Stroke)) OR (CVA)) OR (Acute Cerebrovascular Accident))) AND ((systemic immune-inflammation index) OR (SII)) (Topic)

Table S2. Quality evaluation of the eligible studies with Newcastle–Ottawa scale.

| Study | Selection | | | | Comparability | | Outcome | | |
| --- | --- | --- | --- | --- | --- | --- | --- | --- | --- |
|  | Representative-ness | Selection of  non-exposed | Ascertainment  of exposure | Outcome not present at start | Comparability on most important factors | Comparability on other risk factors | Assessment of outcome | Long enough follow-up (median≥1 week) | Adequacy  (completeness) of follow-up |
| Yiyun Weng 2021 | * | * | * | * | - | - | * | * | - |
| Nan Wang 2022 | * | * | * | * | * | - | * | * | * |
| Yun-Xiang Zhou 2022 | * | * | * | - | - | * | - | * | - |
| Jun Zhao 2023 | * | * | * | - | * | - | * | * | * |
| Ao Qian 2025 | * | * | * | * | - | * | * | * | * |
| Suwen Huang 2024 | * | * | * | - | * | - | * | * | - |
| Zhang Liang 2023 | * | * | * | - | - | * | * | * | * |
| Kadiyan Aierken 2025 | * | * | * | * | * | * | * | * | * |
| Yuan Yang 2022 | * | * | * | * | - | * | * | - | * |
| Yi Yang 2021 | * | * | * | * | * | * | * | * | * |
| Ho Jun Yi 2021 | * | * | * | * | - | * | * | - | * |

*indicates criterion met; - indicates significant of criterion not met.

TableS3. Subgroup analyses based on sample size, study location, age, and SII cutoff values.

| Subgroup | Mortality | | | | mRS score | | | | intracranial hemorrhage | | | |
| --- | --- | --- | --- | --- | --- | --- | --- | --- | --- | --- | --- | --- |
|  | Comparative groups | OR [95%CI] | *P* value | *I*^2^ | Comparative groups | OR [95%CI] | *P* value | *I*^2^ | Comparative groups | OR [95%CI] | *P* value | *I*^2^ |
| **Total** | 11 | 1.58 [1.23-2.02] | 0.0003 | 51% | 13 | 2.03 [1.63, 2.52] | 0.0001 | 69% | 5 | 2.41 [1.59, 3.66] | 0.35 | 9% |
| Sample |  |  |  |  |  |  |  |  |  |  |  |  |
| ≥1000 | 2 | 2.16[1.10-4.24] | 0.02 | 0% | 6 | 1.82 [1.42, 2.32] | 0.004 | 71% | NA | NA | NA | NA |
| ＜1000 | 9 | 1.51 [1.17-1.96] | 0.002 | 53% | 7 | 2.48 [1.57, 3.92] | 0.002 | 72% | 5 | 2.41 [1.59, 3.66] | 0.35 | 9% |
| Country |  |  |  |  |  |  |  |  |  |  |  |  |
| China | 10 | 1.70[1.36-2.11] | ＜0.00001 | 7% | 12 | 2.06 [1.63, 2.61] | 0.0001 | 72% | 5 | 2.41 [1.59, 3.66] | 0.35 | 9% |
| Turkey | 0 | / | / | / | / | / | / | NA | NA | NA | NA | NA |
| America | 1 | / | / | / | / | / | / | / | NA | NA | NA | NA |
| Korea | 0 | / | / | / | 1 | 1.82 [1.16, 2.86] | 0.009 | NA | NA | NA | NA | NA |
| Mean/median age |  |  |  |  |  |  |  |  |  |  |  |  |
| ≥65y | 8 | 1.67[1.17-2.37] | 0.005 | 43% | 8 | 2.35 [1.65, 3.35] | 0.002 | 68% | 5 | 2.41 [1.59, 3.66] | 0.35 | 9% |
| ＜65y | 3 | 1.52[1.02-2.26] | 0.04 | 59% | 5 | 1.73 [1.30, 2.30] | 0.005 | 73% | NA | NA | NA | NA |
| SII cut-off |  |  |  |  |  |  |  |  |  |  |  |  |
| ≥500 | 2 | 1.23[0.80-1.89] | 0.36 | 0% | 10 | 2.18 [1.68, 2.83] | 0.001 | 67% | 3 | 2.50 [1.43, 4.37] | 0.25 | 27% |
| ＜500 | 7 | 1.81[1.41-2.31] | 0.00001 | 0% | 2 | 1.71 [0.84, 3.47] | 0.002 | 81% | NA | NA | NA | NA |
